# Narrow genetic basis for the Australian dingo confirmed through analysis of paternal ancestry

**DOI:** 10.1007/s10709-012-9658-5

**Published:** 2012-05-23

**Authors:** Arman Ardalan, Mattias Oskarsson, Christian Natanaelsson, Alan N. Wilton, Afshin Ahmadian, Peter Savolainen

**Affiliations:** 1 Science for Life Laboratory, Department of Gene Technology, KTH, Royal Institute of Technology, 171 21 Solna, Sweden; 2 School of Biotechnology and Biomolecular Sciences, University of New South Wales, Kensington, NSW 2052 Australia; 3 Ramaciotti Centre for Gene Function Analysis, University of New South Wales, Kensington, NSW 2052 Australia

**Keywords:** Dingo, *Canis familiaris*, New Guinea singing dog, Y-chromosome, Single nucleotide polymorphism (SNP), Protease-mediated allele-specific extension (PrASE), Short interspersed element (SINE)

## Abstract

The dingo (*Canis lupus dingo*) is an iconic animal in the native culture of Australia, but archaeological and molecular records indicate a relatively recent history on the continent. Studies of mitochondrial DNA (mtDNA) imply that the current dingo population was founded by a small population of already tamed dogs from Southeast Asia. However, the maternal genetic data might give a unilateral picture, and the gene pool has yet to be screened for paternal ancestry. We sequenced 14,437 bp of the Y-chromosome (Y-chr) from two dingoes and one New Guinea Singing Dog (NGSD). This positioned dingo and NGSD within the domestic dog Y-chr phylogeny, and produced one haplotype not detected before. With this data, we characterized 47 male dingoes in 30 Y-chr single-nucleotide polymorphism sites using protease-mediated allele-specific extension technology. Only two haplotypes, H3 and H60, were found among the dingoes, at frequencies of 68.1 and 31.9 %, respectively, compared to 27 haplotypes previously established in the domestic dog. While H3 is common among Southeast Asian dogs, H60 was specifically found in dingoes and the NGSD, but was related to Southeast Asian dog Y-chr haplotypes. H3 and H60 were observed exclusively in the western and eastern parts of Australia, respectively, but had a common range in Southeast. Thus, the Y-chr diversity was very low, similar to previous observations for d-loop mtDNA. Overall genetic evidence suggests a very restricted introduction of the first dingoes into Australia, possibly from New Guinea. This study further confirms the dingo as an isolated feral dog.

**Electronic supplementary material:**

The online version of this article (doi:10.1007/s10709-012-9658-5) contains supplementary material, which is available to authorized users.

## Introduction

**Fig. 1:**
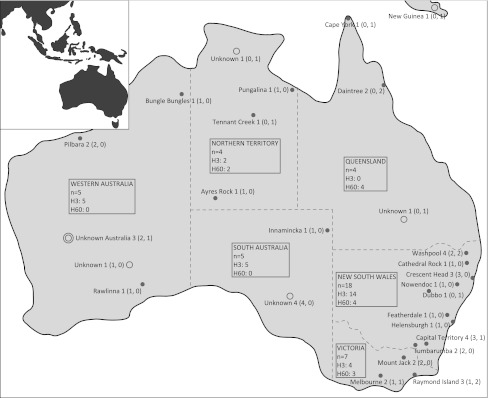
Map of Australia showing sampling locations and information for the sample set used in this study. *Bullet points* exact location; *circle*: approximate area; *double*-*circle* unknown Australian origin

The Australian continent (Fig. 1) was populated approximately 50,000 years before present (ybp) (Mulvaney and Kamminga 1999). Studies of mtDNA, the Y chromosome, and whole genome genetic diversity have revealed that the continent was inhabited by a group of people comprising some of the oldest lineages derived from the African founder types (Hudjashov et al. 2007; Ingman and Gyllensten 2003; Mishmar et al. 2003; Pierson et al. 2006; McEvoy et al. 2010). The Australian population seems to have remained largely isolated after the first entry, and has its closest genetic linkage to the aboriginal populations of New Guinea (Hudjashov et al. 2007; Ingman and Gyllensten 2003; Roberts-Thomson et al. 1996).

The origin of the Australian dingo has generated much interest for the last few centuries (Mulvaney and Kamminga 1999). The earliest undisputed archaeological finding of the dingo has been dated to 3,500 ybp (Smith and Litchfield 2009; Elledge et al. 2006) and was excavated in southern Australia (Gollan 1984; Milham and Thompson 1976). The island of Tasmania was separated from Australia during the sea level rising approximately 12,000 ybp and no traces of dingoes, either contemporary or archaeological, have been found there (Mulvaney and Kamminga 1999). Therefore, the archaeological evidence indicates that the dingoes arrived in Australia between 3,500 and 12,000 ybp. A study of mitochondrial DNA (mtDNA) suggests that dingoes were introduced from the Southeast Asian dog population approximately 4,640–18,100 years, based on the genetic divergence among dingoes (Oskarsson et al*.* 2011).

Based on the apparent correlation in time, it has been suggested that the introduction of the placental dingo to mainland Australia led to the extinction of two major marsupial predators, the Tasmanian tiger (*Thylacinus cynocephalus*) and the Tasmanian devil (*Sarcophilus harrisii*) around 3,500–5,000 ybp (Corbett 1995; Archer 1974) together with the Tasmanian hen (*Gallinula mortierii*) (Baird 1991), which survived thereafter only in the dingo-devoid island of Tasmania. However, some researchers doubt that the dingo alone could have such extensive ecological impacts (Johnson and Wroe 2003; Paddle 2002).

As the sole large eutherian mammal present before modern times in Australia, the dingo seems to have been introduced by man into this continent. However, how and why the feral dingo was imported into Australia is not entirely clear. Studies of morphology suggest a closer resemblance to South Asian than to other dogs (Corbett 1995). The possibility of an origin from India has been suggested since the dingo shares similarities in skeletal anatomy with Indian Pariah dogs and wolves (*Canis lupus*) (Gollan 1985; Clutton-Brock 1995). However, these studies did not include samples from parts of the world other than India, e.g. Southeast Asia, and were based on very few samples.

There are clear similarities, but also differences, between the New Guinea Singing Dog (NGSD) and the dingo, in terms of behaviour, morphology, and anatomy (Koler-Matznick et al*.* 2003). Further, studies of mtDNA and the Major Histocompatibility Complex have shown extensive haplotype sharing between these two populations (Oskarsson et al*.* 2011; Runstadler et al*.* 2006; Savolainen et al*.* 2004). One study of mtDNA also strongly indicated that the Australian dingo originated from East Asian domestic dogs, because the sole mtDNA founder haplotype detected for the Australian dingo population was a dog haplotype commonly found throughout East Asia, and almost exclusive to this region (Savolainen et al*.* 2004). In that study, the possibility that dingoes arrived as part of the Neolithic culture with the Austronesian expansion was suggested. However, a later study of dingoes and domestic dogs in Polynesia and Island Southeast Asia indicated that dogs were introduced into Australia via mainland Southeast Asia before the spread of the Neolithic, possibly through contacts with New Guinea, and not with the Austronesian expansion through Taiwan (Oskarsson et al*.* 2011). Microsatellite data from one study revealed haplotype sharing between Bali Street Dogs and Australian dingoes (Irion et al*.* 2005).

In relationship with humans, the dingo gained an important position in the Australian native culture, as a representation of man in the animal world and a symbolic icon of the indigenous spirituality (Smith and Litchfield 2009). Dingoes are primarily wild, but they appear to have been in a semi-domestic relationship with aboriginal inhabitants of Australia in several cases (Smith and Litchfield 2009). Compared to wolves, dingoes can more effectively connect to human social cues. Their performance in comprehending human gestures lies between wolves and dogs, most probably due to their early domestic history and origin from domestic dog populations (Smith and Litchfield 2010). The “wildness” of the dingo is possibly a product of more than 3,000 years of feral life in Australia, or a not fully domesticated state (in the modern sense) of the founder animals, and possibly a combination of both.

Genetic studies indicate low diversity for the dingo population. A study of microsatellite allelic variability revealed much lower genetic diversity among Australian dingoes than among domestic dogs (Wilton et al*.* 1999). Similarly, the mtDNA variation among dingoes is considerably lower than among domestic dogs: a single haplotype, A29, was found in 83 % (192/232) of the samples, and all other haplotypes were separated from A29 by a single substitution, and almost all were unique to dingoes, and exclusive to their locations (Oskarsson et al*.* 2011; Savolainen et al*.* 2004). Therefore, the dingo founders probably carried only a single mtDNA haplotype that subsequently evolved into several derived haplotypes. This implies that the dingo population was founded from a very small population of dogs, or from a population that had undergone numerous bottlenecks dramatically reducing genetic diversity (Oskarsson et al*.* 2011; Savolainen et al*.* 2004; Wilton et al*.* 1999).

In nature, males and females of many animal species may not contribute equally to the gene pool of a newly founded population. Particularly in mammals, males are often the dispersing sex, and the natural migration pattern is often male-biased (Greenwood 1980). This was shown to be the case in several canid species (Macdonald and Sillero-Zubiri 2004), as well as in Indian free-ranging dogs (Pal et al*.* 1998), and in dingoes (Thomson et al*.* 1992). Considering the otherwise isolated status of the Australian fauna, assistance of humans in the spread of dingoes into Australia seems to be the most likely scenario. However, there is also a possibility that this spread took place through natural processes. In that case, a richer paternal ancestry could be anticipated in comparison with the female founder stock. Also in the case of human introduction of the dingo, mtDNA data may give a sexually biased picture of the history of dingoes. Thus, we studied the Y-chromosome (Y-chr) DNA diversity among dingoes in order to resolve the foundation event(s) forming today’s dingo populations in Australia, and compared these data with variation in maternally inherited mtDNA.

**Fig. 2:**
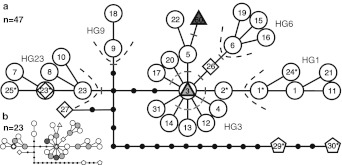
The most parsimonious tree showing the phylogeny of sequences from non-homologous regions of the Y-chr adopted from (Ding et al*.* 2011). *Circles* represent dog haplotypes; *triangles* represent dingo haplotypes; *squares* represent wolf haplotypes; *pentagons* represent coyote haplotypes; *black bullet points* represent assumed intermediary haplotypes. The *lines* between haplotypes represent one mutation steps. *Orange* represents unique haplotypes; *green* represents shared haplotypes. **a** SNP analysis procedure and results for 47 dingoes: two haplotypes were found, one unique and one shared. *Colored dashes* show SNPs screened for the diagnosis of haplogroups and haplotypes in the first (*red*) and second (*blue*) assays. For SNP locations see Table S2. *Dashed lines* indicate separate haplogroups. Haplotype H31 was not available at the time of the analysis, and therefore was not taken into analysis. **b** Illustration of the Y-chr diversity for ASY (Asia South of Yangtze River): 13 haplotypes were found among 23 dogs, two unique and 11 shared; data from Ding et al*.* (2011). (Color figure online)

In a previous study, 14,437 bp of non-homologous Y-chr DNA was sequenced in 151 domestic dogs from throughout the world, 12 Eurasian wolves, and two North American coyotes (*Canis latrans*) (Natanaelsson et al. 2006; Ding et al. 2011). Consequently, 32 haplotypes were defined: 28 in domestic dogs (one shared with wolf), two in wolves, and two in coyotes (Fig. 2a). These haplotypes were distinguished by 50 diagnostic single nucleotide polymorphism (SNP) positions, 30 of which were polymorphic in domestic dogs (Table S2). The dog haplotypes were arranged in five groups of more closely related haplotypes, or haplogroups (Fig. 2a) (Natanaelsson et al. 2006; Ding et al. 2011).

In this study, we sequenced this region for two dingoes and one NGSD to establish whether dingo sequences group within the general dog Y-chr phylogeny, to identify potential new haplotypes, and to detect possible corresponding SNPs. Then we screened Y-chr diversity for 47 male dingoes (Table S1) from across Australia (Fig. 1) by SNP analysis. We used these data to infer the origin and dispersal history of the dingo paternal lineages.

## Materials and methods

### Layout and analysis

#### Samples

Blood samples were collected from 47 captive and wild unrelated male dingoes at different locations in Australia (Table S1, Fig. 1). Effort was taken to sample dingoes with as little admixture with domestic dogs as possible based on analyses of dingo diagnostic microsatellites as well as phenotype, described in Wilton (2001) and Wilton et al. (1999).

#### DNA extraction

DNA was obtained from the EDTA-treated blood samples through proteinase K treatment, chloroform extraction and ethanol precipitation, as described in Wilton (2001).

#### Experiment design

We sequenced 14,437 bp of non-homologous Y-chr DNA (Natanaelsson et al*.* 2006) for two dingoes and one NGSD in order to determine whether dingo sequences fall in the canid phylogeny for Y-chr (Ding et al*.* 2011), as well as to identify and allocate possibly specific dingo Y-chr haplotype(s) in this phylogeny. We then used an automated microarray-based SNP genotyping assay, protease-mediated allele-specific extension (PrASE) (Hultin et al*.* 2005), for screening polymorphisms in a multiplex amplification of diagnostic SNP sites for 47 male dingoes, including two of the sequenced samples. The method has been shown to be as accurate as pyrosequencing (Käller et al. 2006), and the set-up would allow inspection of the whole Y-chr phylogeny previously available (Ding et al*.* 2011).

### DNA sequencing

#### PCR amplification

The PCR amplification was performed in a nested configuration with outer and inner primer pairs in order to increase sensitivity and specificity. Reactions for 18 separate Y-chr fragments were run in 50 μl volume, as described in a previous study (Natanaelsson et al*.* 2006). Extracted DNA was used at 1 μl volume for template. The outer PCR reaction was run in a Thermo Hybaid MBS 0.2 S (Thermo Electron Corporation, Waltham, MA): pre-denaturation at 94 °C for 2 min, followed by 15 cycles of denaturation at 94 °C for 30 s, primer annealing at 55 °C for 30 s, and extension at 72 °C for 3 min, finished by a final extension step at 72 °C for 10 min. The inner PCR reaction was identical except for that 1 μl of outer amplification product was used as template, and the inner PCR program consisted of 35 cycles.

#### Sanger sequencing

The cycle sequencing reaction was performed in 20 μl reaction volume for each of the 18 amplified fragments, using the same reagents and cycle sequencing program as Natanaelsson et al*.* did. The cycle sequencing products were ethanol precipitated and sequenced on an ABI 3700 according to the manufacturers instructions (Applied Biosystems). DNA sequences were edited and assembled into contigs using the program Sequencher 4.1 (Gene Codes Corporation, Ann Arbor, MI).

### SNP analysis

#### Approach description

One new Y-chr haplotype found through DNA sequencing in both dingoes and NGSDs and 27 dog haplotypes from previous studies (Ding et al*.* 2011), defined by 29 diagnostic SNPs, were assayed by high-throughput SNP screening of dingo samples. SNPs were detected and compared to haplotype sequences (Table S2) allowing clustering into haplogroups and haplotypes (Natanaelsson et al. 2006; Ding et al. 2011). The systematic assignment of haplotypes by SNPs provided an internal control for the genotyping. The Y-chr phylogeny constructed (Fig. 2a) represents the most parsimonious connections between the haplotypes without homoplasy in polymorphic nucleotide positions (Ding et al*.* 2011). Thus, complete precision in genotyping by SNP sites was achieved.

#### Assay strategy

Diagnostic SNPs were asayed in two separate rounds of analysis to assign haplogroups on the first step of screening and haplotypes on the second. Haplogroup SNPs at positions defining haplogroups 2, 5, 6, 9, 23, wolf H26, and dingo H60 (Table S2, Fig. 2a) were first screened for all samples to locate them within dog haplogroups or wolf/dingo sub-haplogroups. Based on the results from this rough positioning, haplotype SNPs were screened to determine the actual haplotype. Since all samples fell into either haplogroup 3 or the dingo sub-haplogroup on the first assay, only SNPs at positions defining haplotypes 4, 12, 13, 14, 17, 20 were used in the second assay (Table S2, Fig. 2a) to analyze the samples in question within haplogroup 3. All these samples were shown to belong to haplotype H3 (Fig. 2a).

#### PrASE amplification

SNP sites were amplified in Polymerase Chain Reactions (PCRs) on a Thermo Hybaid MBS 0.2 S (Thermo Electron Corporation) in short fragments. Nested amplification was devised using two primer pairs of outer and inner annealing sites for each fragment (Table S3) in order to maximize specificity of the products. Each of the outer and inner PCRs were run in multiplexed reactions, so that all SNP loci could be amplified in a single reaction for each individual, considerably reducing complexity of the analyses.

The PCR mix for the 25 μl outer reaction was as follows: 0.5 U Platinum taq polymerase, PCR buffer 1X, dNTPs 0.2 mM, Mg^2+^ 2 mM, forward primer 0.2 mM, reverse primer 0.2 mM, 1 μl template DNA extract, 15.9 μl ddH2O. The products from the outer reaction were taken to the inner reaction as template DNA.

The inner PCR contained a single biotin-labeled primer (Table S3) at high concentration, and a pair of normal primers for each SNP, one of them tagged with a short freely-hanging handle designed to anneal to the biotinylated primer. Due to the relatively high cost of primer biotinylation, this strategy was used to incorporate biotin in the final products using a universal biotinylated oligonucleotide. The biotin molecule enables immobilization on streptavidin-coated magnetic beads, to keep the products on solid phase during the washing-out process. The PCR mix for the 50 μl inner reaction was as follows: 1 U Platinum Taq DNA polymerase (Invitrogen), PCR buffer 1X, dNTPs 0.2 mM, Mg^2+^ 2 mM, normal primer 0.1 mM, tagged primer 0.025 mM, biotinylated primer 0.2 mM, 1 μl outer PCR product, 33.55 μl ddH2O.

The PCR programme for the reactions was as follows: initial denaturation at 94 °C for 5 min; 35 cycles of denaturation at 94 °C for 30 s, annealing at 55 °C for 90 s (outer) or 45 °C for 1 min (inner), and extension at 72 °C for 40 s; and a final extension at 72 °C for 10 min.

#### PrASE reaction

The full protocol for automated multiplex PrASE reaction is described in Hultin et al*.* (2005). In brief, allele-specific extension primers encompassing unique tag sequences (Table S3) were used for the hybridisation of PrASE products to a generic tag array. In the PrASE reaction, a dNTP mix containing partially Cy5-labeled nucleotides was used to enable laser detection.

#### SNP screening

The slides were visualized using a scanner (Agilent Technologies, Palo Alto, CA) and the images were compiled using Feature Extraction software (version A.6.1.1, Agilent Technologies). The microchips on the slides were exposed to red laser of 635 nm wavelength to detect and measure signals from the spots. The spot signal intensity was assessed using a GenePix Pro 5.0 scanner (Molecular Devices, Sunnyvale, CA), and the results were analyzed in R version 2.8.1 (R Foundation for Statistical Computing, Vienna, Austria) to assign genotypes.

## Results

We sequenced two dingoes and one New Guinea Singing Dog for the 14,437 bp of Y-chr DNA in order to place dingoes in the canid Y-chr phylogeny (Ding et al*.* 2011). We found two haplotypes, as defined by substitutions: H3 in one dingo and H60 in the other dingo and in the NGSD. The H60 sequence from the dingo also included an insertion of 300 bp in position 1,015 of fragment 20 (Table S2). It had a polyadenylation signal (AATAAA) and a poly-A stretch in its 3′ tail, indicating a short interspersed element (SINE) (Singer 1982). This SINE was almost identical to several dog and wolf GenBank records, but is reported here for the first time from canine Y-chr. The sequence containing this SINE was named H60SINE (GenBank accession numbers: JQ723489 for H60, and JQ723490 for H60SINE). Haplotype H3 was previously found in domestic dogs from East Asia (n = 9) and North Europe (n = 2) (Ding et al*.* 2011). H60 is a novel and not previously reported haplotype, but is separated by one substitutional step from H5 (Fig. 2a) which has been found in domestic dogs exclusively from East Asia (Cambodia, China, Japan and Siberia) (Ding et al*.* 2011). Thus, the sequence analysis places dingo Y-chr haplotypes among dogs in the canid Y-chr phylogeny, and establishes the East Asian ties previously inferred from the mtDNA data (Savolainen et al*.* 2004). From the three sequenced samples, one dingo with haplotype H3 and the NGSD with haplotype H60 were also genotyped by SNP analysis, identifying the same haplotypes.

We screened Y-chr diversity among dingoes by an SNP assay using 29 of the polymorphic sites previously detected among dogs (Ding et al*.* 2011), and the newly-detected SNP position defining the novel haplotype H60 of the dingo/NGSD (Table S2, Fig. 2a). An additional SNP site detected in dogs by Ding et al*.* was not available at the time of our analysis. Samples from 47 dingoes across Australia (Fig. 1) were investigated, and all were found to have either of the two haplotypes identified by DNA sequencing. Thirty two samples (68.1 % of the individuals) had H3 and 15 samples (31.9 %) had the novel haplotype H60. Thus, only two haplotypes were found among our total sample of 47 dingoes. This is a much lower diversity than observed in the previously investigated dog populations (Ding et al*.* 2011). In comparison, Asian dogs from south of the Yangtze river (a region denoted “Asia South of Yangtze River”—ASY), the probable source population for the dingo, had 13 Y-chr haplotypes among 23 investigated individuals (Fig. 2b) (Ding et al*.* 2011).

Haplotypes H3 and H60 were not evenly distributed across Australia. In the best-sampled regions, New South Wales and Victoria, in the most densely populated southeast part of Australia, both H3 and H60 were present at frequencies of 72 and 28 %, respectively (Fig. 1). However, in Queensland in the northeast part of Australia only H60 was found, and all through South Australia and Western Australia only H3 was present. With the relatively small sample sizes from these regions (10 from the south and west and 4 from the northeast), a complete absence of H3 from the northeast and of H60 from the southern and western parts of Australia cannot be positively established. Nonetheless, there was a clear difference in frequencies between the two parts of Australia. The frequency of H60 was significantly below 25.9 % in south and west and above 47.3 % in the northeast, and correspondingly the frequency of H3 was significantly below 52.7 % in the northeast and above 74.1 % in south and west (*P* < 0.05). Thus, a pattern of haplotype distribution based on geography was observed, approximately concordant with the climatic regions of Australia. A similar structure is also reflected in the mtDNA data for which the founder haplotype is present all over Australia, and the derived haplotypes mostly follow a local distribution (Oskarsson et al*.* 2011; Savolainen et al*.* 2004).

Haplotype H60, which was the only haplotype found in northeast Australia, was also found in the NGSD. This may represent a relationship between NGSDs and dingoes, as previously indicated by mtDNA data (Savolainen et al. 2004; Oskarsson et al. 2011), as well as a connection between New Guinea and northeast Australia, suggesting a northeastern route for the entry of dingoes. However, the geographic pattern of haplotype distribution with H3 being the dominant haplotype in the west may suggest an independent introduction from the northwest, but it may also be a product of random genetic drift.

## Discussion

While mtDNA sequence data has indicated a narrow ancestry and effective subsequent isolation on the maternal side for Australian dingoes, the formation and history of the dingo population in Australia may be very different considering paternal ancestry. If more male founders would be found scattered throughout Australia this would challenge the scenario of a highly bottlenecked and relatively recent foundation of Australian dingoes from the southeast Asian dog population. However, we show that only two haplotypes of the Y-chr were present in a relatively well-distributed sample of 47 pure dingoes throughout Australia. The Y-chr SNP variation observed here was similar to that of the mtDNA data for which all 211 dingoes seemingly had mtDNA haplotypes originating from a single founder haplotype, A29 (Savolainen et al*.* 2004). Thus, the bottleneck indicated by the mtDNA data was not caused by a lower contribution of females than males to the founding gene pool due to a male-biased dispersal or other mechanisms. Instead, the paternal gene pool of the Australian dingo also reflects a highly bottlenecked ancestry.

In comparison, more than 25 distinct mtDNA haplotypes are present in today’s Indonesia and New Guinea. Even purebred European dog breeds that have been exposed to extreme artificial selection usually carry several mtDNA haplotypes. For instance, 6 founder mtDNA haplotypes were observed in 27 German Shepherd dogs (Angleby and Savolainen 2005). Similarly, two Y-chr haplotypes among 47 Australian dingoes represents a very low diversity compared to 13 haplotypes found among 23 dogs in the nearby ASY (Fig. 2b). Thus, we infer that the ancestry of the dingo was strictly limited, likely based on only a few individuals, at one or only few occasions. Furthermore, no new haplotypes have been introduced after the initial introduction implying effective isolation of the dingo population in Australia once it was established.

Since southern East Asia has the highest genetic diversity among dogs for both mtDNA (Pang et al. 2009; Ardalan et al. 2011) and Y-chr DNA (Ding et al*.* 2011), the limited genetic basis of the dingo population of Australia indicates a very restricted gene flow. Obviously, the gene flow from southern East Asia has been much more efficient to the rest of Eurasia and Africa (Ardalan et al*.* 2011) which is connected by land, than to Australia which is relatively close but separated by several “island hops”.

There is a high degree of reciprocal sharing of the gene pools of the Australian dingoes and dogs in New Guinea. One of the two dingo Ychr haplotypes, H60, was found in the single NGSD analyzed, and the full genetic diversity for mtDNA among dingoes is shared with New Guinean dogs, including NGSDs, in the sense that the single founder haplotype for the dingoes (A29) is present at a high proportion (20 %) in New Guinea (Oskarsson et al*.* 2011). Furthermore, all Dog Leucocyte Antigen (DLA) alleles found in NGSDs were shared with the Australian dingo (Runstadler et al*.* 2006).

This extensive genetic sharing indicates an origin of dingoes via New Guinea. The fact that all dingoes share a single mtDNA founder haplotype while there are several mtDNA haplotypes present in today’s Indonesia and New Guinea also suggests that this was the only introduction route of dogs to Australia, since introductions of dogs from multiple regions into Australia should have brought several different mtDNA founder haplotypes.

However, while both haplotype H3 and H60 were found in the southeast part of Australia, H3 was absent in the northeast and H60 in the west, reflecting a clear difference in the frequencies of H3 and H60 between these regions. This pattern suggests the possibility of a separate introduction in the west of dogs carrying H3. Microsatellite data showed 75 % of Australian dingoes to share alleles exclusively with Bali street dogs, as compared to 28 dog breeds (Irion et al*.* 2005). There was also considerable sharing of DLA alleles between Bali street dogs and dingoes (Runstadler et al*.* 2006). If H3 was introduced from the Bali region in northwest, the distribution of the two Y-chr haplotypes across Australia could be caused by the barrier formed by the central Australian desert. However, since all dingoes have mtDNA haplotypes deriving from A29, a separate origin of H3 would imply that A29 has been introduced twice as the single mtDNA haplotype, in the northwest as well as the northeast. This does not seem likely given that several mtDNA haplotypes are present in today’s Indonesia and New Guinea and the frequency of A29 in Bali was only 2 % (Oskarsson et al*.* 2011). A more simple explanation is that the Ychr distribution-pattern is the result of random genetic drift after introduction of both H3 and H60 from the northeast, in line with a single origin from New Guinea. Studies with higher coverage of samples from across Australia as well as representation from Island Southeast Asia may give more information about the distribution of H3 and H60 in Australia and its surroundings, shedding more light on whether dingoes were introduced only once or several times, and by which routes this occurred.

## Conclusions

With this study we show the paternal ancestry of the Australian dingo population to be as narrow as previously demonstrated for the maternal side (Savolainen et al*.* 2004). The dingo population was likely based on introduction of only a few individuals, at one or only few occasions. A high degree of sharing of the gene pools of the Australian dingoes and dogs in New Guinea indicates that the dingoes were introduced, probably exclusively, via New Guinea. This study provides further proof that the dingo population has remained strictly isolated from nearby areas since it was established in Australia (Oskarsson et al. 2011; Savolainen et al. 2004; Wilton et al. 1999; Irion et al. 2005). Factors responsible for this isolation remain to be resolved in future multidisciplinary studies.

## Electronic supplementary material

Below is the link to the electronic supplementary material.

Table S1: Total sample list, with haplotype results, status of the animal, area of sampling and origination, and lineage information. Two samples, shown in green, were analyzed by DNA sequencing, in addition to SNP-genotyping. The sample shown in yellow was only analyzed by DNA-sequencing, and was therefore not included in the sample set calculations. It also carried a SINE insertion. (XLS 19 kb)

Table S2. Alignment of Y-chr haplotypes, showing fragments and positions of the polymorphic sites plotted against reference (in yellow) (Natanaelsson et al*.* 2006). Only sites with substitutions are considered as variable to define haplotypes (HT). SNP sites screened on the first assay (haplogrouping) are shown in red, and those on the second assay (haplotyping) in blue, as in Fig. 2. Haplotypes from wolf, coyote and dingo/NGSD are shown in grey, blue, and orange, respectively. Haplotypes with duplication (i.e. from an individual that yields equal proportions of both reference and mutant alleles) are shown by *. A 300 bp insertion in dingo H60 is shown by +. Equivalent haplotypes differing by indels (Indel HT) are shown by letters a, b, and c. SNP positions non-existing in the reference are shown by/and a number. The sequenced fragment 12 showed no mutation, and is therefore unrepresented by SNPs. (XLS 77 kb)

Table S3. Primers used in the first and second assays of the PrASE procedure. Respectively from left, columns represent round of screening, polymorphic fragment, polymorphic position, SNP alleles, haplotype defined by the SNP, diagnosed haplogroup or haplotype, outer primers, inner primers, and tagged PrASE primers. The biotinylated primer is given separately. (XLS 32 kb)
